# Respiratory mechanics and lung stress/strain in children with acute respiratory distress syndrome

**DOI:** 10.1186/s13613-016-0113-0

**Published:** 2016-02-05

**Authors:** Davide Chiumello, Giovanna Chidini, Edoardo Calderini, Andrea Colombo, Francesco Crimella, Matteo Brioni

**Affiliations:** Dipartimento di Anestesia, Rianimazione (Intensiva e Subintensiva) e Terapia del Dolore, Fondazione IRCCS Ca’ Granda - Ospedale Maggiore Policlinico, Via F. Sforza 35, Milan, Italy; Dipartimento di Fisiopatologia Medico-Chirurgica e dei Trapianti, Università degli Studi di Milano, Milan, Italy; Terapia Intensiva Pediatrica, Dipartimento di Anestesia e Rianimazione, Fondazione IRCCS Ca’ Granda - Ospedale Maggiore Policlinico, Milan, Italy

**Keywords:** Acute respiratory distress syndrome, PEEP, Lung stress, Lung strain, Functional residual capacity, Ventilator-induced lung injury, Tidal volume

## Abstract

**Background:**

In sedated and paralyzed children with acute respiratory failure, the compliance of respiratory system and functional residual capacity were significantly reduced compared with healthy subjects. However, no major studies in children with ARDS have investigated the role of different levels of PEEP and tidal volume on the partitioned respiratory mechanic (lung and chest wall), stress (transpulmonary pressure) and strain (inflated volume above the functional residual capacity).

**Methods:**

The end-expiratory lung volume was measured using a simplified closed circuit helium dilution method. During an inspiratory and expiratory pause, the airway and esophageal pressure were measured. Transpulmonary pressure was computed as the difference between airway and esophageal pressure.

**Results:**

Ten intubated sedated paralyzed healthy children and ten children with ARDS underwent a PEEP trial (4 and 12 cmH_2_O) with a tidal volume of 8, 10 and 12 ml/kg_IBW_. The two groups were comparable for age and BMI (2.5 [1.0–5.5] vs 3.0 [1.7–7.2] years and 15.1 ± 2.4 vs 15.3 ± 3.0 kg/m^2^). The functional residual capacity in ARDS patients was significantly lower as compared to the control group (10.4 [9.1–14.3] vs 16.6 [11.7–24.6] ml/kg, *p* = 0.04). The ARDS patients had a significantly lower respiratory system and lung compliance as compared to control subjects (9.9 ± 5.0 vs 17.8 ± 6.5, 9.3 ± 4.9 vs 16.9 ± 4.1 at 4 cmH_2_O of PEEP and 11.7 ± 5.8 vs 23.7 ± 6.8, 10.0 ± 4.9 vs 23.4 ± 7.5 at 12 cmH_2_O of PEEP). The compliance of the chest wall was similar in both groups (76.7 ± 30.2 vs 94.4 ± 76.4 and 92.6 ± 65.3 vs 90.0 ± 61.7 at 4 and 12 cmH_2_O of PEEP). The lung stress and strain were significantly higher in ARDS patients as compared to control subjects and were poorly related to airway pressure and tidal volume normalized for body weight.

**Conclusions:**

Airway pressures and tidal volume normalized to body weight are poor surrogates for lung stress and strain in mild pediatric ARDS.

*Trial registration*: Clinialtrials.gov NCT02036801. Registered 13 January 2014

**Electronic supplementary material:**

The online version of this article (doi:10.1186/s13613-016-0113-0) contains supplementary material, which is available to authorized users.

## Background

Mechanical ventilation is routinely applied for different reasons in up to 64 % of children admitted to pediatric intensive care units [1]. One of the most frequent applications is for the acute respiratory distress syndrome (ARDS) which has been defined more than 20 years ago and recently revised [2, 3]. ARDS has been reported to affect from 2.9 up to 12.8 patients per 100.000 children per year [4, 5] with an associated mortality ranging between 18 and 35 % [4–7]. Children with ARDS are frequently managed with a tidal volume, between 9 and 10 ml/kg of predicted body weight [4] and mean PEEP level between 6 and 10 cmH_2_O [6, 8]. Contrary to adult ARDS patients, in which several randomized clinical studies have shown that the application of a lung protective ventilation (low tidal volume and high PEEP levels) ameliorated the final outcome [9, 10], in children with ARDS only few clinical studies have suggested a benefit of this strategy [11–13]. In addition, there are conflicting reports about the relationship between size of tidal volume and the outcome [9, 11, 14, 15].

In sedated, paralyzed children with non-cardiogenic acute respiratory failure, the compliance of respiratory system and functional residual capacity were significantly lower than in healthy subjects [16–18]. The application of PEEP was able to increase the compliance of respiratory system and to normalize the functional residual capacity in the majority of the patients [17, 18]. However, no major studies in children with ARDS have investigated the role of different levels of PEEP and tidal volume on the partitioned respiratory mechanic (lung and chest wall), stress (i.e., the transpulmonary pressure at end inspiration) and strain (the change in volume to the functional residual capacity) [19, 20]. Like in adult ARDS patients, an estimate of how much respiratory system is impaired should be fundamental for optimizing the ventilatory strategy (i.e., to minimize the ventilation induced lung injury). The aim of this study was to evaluate in children with ARDS how the respiratory mechanics and stress/strain are affected compared with normal subjects.

## Methods

### Study population

The study was approved by the ethics committee of the Fondazione IRCCS “Ospedale Maggiore Policlinico Mangiagalli Regina Elena,” Milano. Before enrollment, written informed consent was obtained by the family for each patient (Clinical trials NCT02036801). Patients were enrolled from April 2009 to April 2014. The study population comprised two groups: first group—ten patients with ARDS [2, 3], and second group—ten control subjects after elective surgery or neurologic diseases without acute respiratory failure, cardiac disease and any signs of upper respiratory infections.

### Study design

All patients were intubated with a cuffed endotracheal tube, sedated, paralyzed and kept in supine position. Anesthesia and paralysis were maintained with midazolam 2 mcg/kg/min, fentanyl 1 mcg/kg/h and cisatracurium 2 mcg/kg/min. All measurements were taken after inflating the endotracheal tube cuff to prevent leaks up to 40 cmH_2_O.

A computer-driven protocol of ventilator setting was used [19]. The sequence started with the measurement of functional residual capacity. After this measurement, the ventilator applied 60 consecutive breaths with a tidal volume of 8, 10 and 12 ml/kg of ideal body weight at 4 and 12 cmH_2_O of PEEP. For more details, see Additional file 1: Figure S1. In order to standardize the lung volume history, before the measurement of functional residual capacity and changes in PEEP, a recruitment maneuver obtained by increasing the tidal volume to reach 35 cmH_2_O starting from 5 cmH_2_O of PEEP with a respiratory rate of 10 bpm was performed

### Measurements

The respiratory flow rate was measured with a heated pneumotachograph (Fleisch No. 2, Fleisch). Airway pressure was measured proximally to the endotracheal tube with a dedicated pressure transducer (MPX 2010 DP, Motorola). Esophageal pressure was measured with a radio-opaque esophageal balloon (length 40 cm, diameter 6 Fr) (CareFusion, Linda, USA) inflated with 0.2–0.3 ml of air connected to a pressure transducer. All traces were sampled at 100 Hz and processed on a dedicated data acquisition system (Colligo and Computo, www.elekton.it). To ensure the correct position of the catheter, the esophageal balloon was positioned in the stomach to check the presence of positive deflection. Then, it was retracted until it reached the lower third of the esophagus; in this position, an inspiratory occlusion was made to check for concordant changes in airway and esophageal pressure [19, 21].

The functional residual capacity and end-expiratory lung volume were measured using a simplified closed circuit helium dilution method by inflating the respiratory system with 0.5–1 l of a mixture of helium and oxygen [19]. The predicted functional residual capacity was estimated according to Sivan et al. [17].

During an inspiratory and expiratory pause, the airway and esophageal pressure were measured. Transpulmonary pressure was computed as the difference between airway and esophageal pressure.

The respiratory system, lung and chest wall compliance were computed according to the following formula [22]:

- Respiratory system compliance (C, rs) (ml/cmH_2_O) = tidal volume/(airway pressure at end inspiration − airway pressure at PEEP)
- Lung compliance (C, l) (ml/cmH_2_O) = tidal volume/(transpulmonary pressure at end inspiration − transpulmonary pressure at PEEP)
- Chest wall compliance (C, cw) (ml/cmH_2_O) = tidal volume/(esophageal pressure at end inspiration − esophageal pressure at PEEP)

Stress and strain were computed as the delta transpulmonary pressure measured from end inspiration to atmospheric pressure and as the ratio between the inflation volume (tidal volume plus the volume due to PEEP) and functional residual capacity [19]. The specific lung elastance was computed as the ratio between the stress and strain.

Airway driving pressure was computed as airway pressure at end inspiration–airway pressure at PEEP

Body weight and height were measured the day of the study by a dedicated balance and by a tape.

### Statistical analysis

Data are reported as mean ± SD or as median [IQ], unless otherwise specified, as appropriate. Statistical significance was defined as *p* < 0.05. Baseline and physiologic variables were compared by Student’s *t* test for variables that were normally distributed and by Mann–Whitney *U* test for variables that were not normally distributed and by Chi-square test for qualitative variables. Linear regression was used to model the relationship between variables and to describe linear segment of the volume/pressure curve. Three-way ANOVA was used to describe the effects of the presence of the disease, the level of PEEP and tidal volume. Bonferroni’s *t* test was employed to correct for multiple comparisons. Power least squares fitting was used to describe the shape of the volume/pressure curve shape. Analysis was performed using SigmaPlot software, version 12.0 (Systat, Chicago, IL).

## Results

The baseline characteristics are reported in Table 1. A total of 20 subjects were enrolled (10 in each group). Age, height, weight and body mass index were similar in the two groups. Patients with ARDS had significantly lower oxygenation, higher airway plateau pressure and higher level of PEEP as compared to control subjects. The functional residual capacity in ARDS patients was significantly lower than predicted (162 ± 68 ml vs 344 ± 152 ml, *p* < 0.01) but was closer in the control group (282 ± 107 vs 382 ± 112 ml, *p* = 0.07). The functional residual capacity was related to the age of the patients in ARDS and in the control group (*r*^2^ = 0.71, *p* < 0.05; *r*^2^ = 0.49, *p* < 0.05) (see Additional file 1: Figures S2, S3).

**Table 1:** Baseline characteristics

Characteristic	ARDS patients (*n* = 10)	Control patients (*n* = 10)	*p* value
Age (years)	2.5 [1.0–5.5]	3.0 [1.7–7.2]	0.62
Male sex, no. of patients (%)	7 (70)	4 (40)	0.37
Height (cm)	99.5 ± 22.2	105.7 ± 14.7	0.47
Weight (kg)	12.7 [10.0–20.2]	15.0 [14.7–18.0]	0.16
Body mass index (kg/m^2^)	15.1 ± 2.4	15.3 ± 3.0	0.88
*V*_T_ (ml)	120.0 [98.7–182.5]	145.0 [127.5–162.5]	0.43
*V*_T_/IBW (ml/kg)	9.1 [7.8–10.0]	9.7 [8.5–10.2]	0.54
Days of ventilation before study	2 [1–4]	0 [0–1]	0.04
ARDS classification, no (%)			
Mild	6 (60)		
Moderate	2 (20)		
Severe	2 (20)		
Respiratory rate (bpm)	27 ± 7	22 ± 5	0.09
Minute ventilation (l/min)	3.4 ± 1.1	3.2 ± 0.8	0.64
Airway plateau pressure (cmH_2_O)	24.2 ± 4.0	15.1 ± 3.1	<0.001
PEEP (cmH_2_O)	8.4 ± 2.3	3.5 ± 1.8	<0.001
PaO_2_/FiO_2_ ratio	206 ± 86	389 ± 73	<0.001
PaCo_2_ (mmHg)	44.0 [37.2–52.5]	40.5 [38.0–45.5]	0.40
FRC (ml)	162.2 ± 67.6	288.1 ± 107.3	0.006
FRC (ml/kg)	10.4 [9.1–14.3]	16.6 [11.7–24.6]	0.04
Admission diagnosis, no (%)			0.004
Sepsis	2 (20)	0	
Infection	4 (40)	0	
ARDS ndd	3 (30)	0	
Post-surgery	0	4 (40)	
Neurological diseases	0	4 (40)	
Other	1 (10)	2 (20)	

Data presented as mean values (SD) or as median [IQ] or as number of subjects (%) as appropriate

*V*_*T*_ tidal volume, *IBW* ideal body weight, *ARDS* acute respiratory distress syndrome, *PEEP* positive end-expiratory pressure, *PaO*_*2*_ */FiO*_*2*_ ratio of partial pressure of arterial oxygen and fraction of inspired oxygen, *PaCO*_*2*_ partial pressure of arterial carbon dioxide, *FRC* functional residual capacity

### Partitioned respiratory mechanics and end-expiratory lung volume: effect of PEEP and tidal volume

**Fig. 1:**
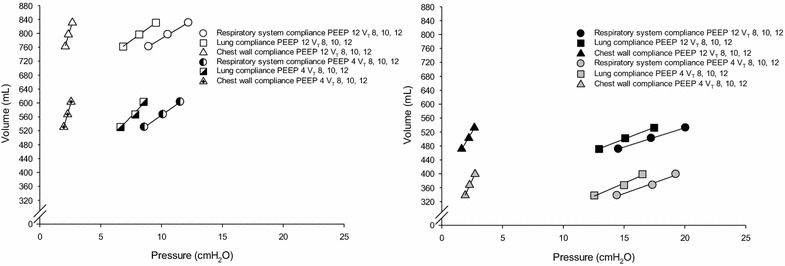
Pressure volume curve of respiratory system, lung and chest wall in control (*left panel*) and ARDS patients (*right panel*)

In both groups, the airway plateau pressure significantly increased by increasing the tidal volume and the PEEP (Table 2). ARDS patients had a significantly lower compliance of the respiratory system and compliance of the lung than controls; both of them were not affected by the PEEP or tidal volume. Compliance of the chest wall was similar in both groups and did not change with PEEP or tidal volume. In Fig. 1 are shown the pressure volume curves of respiratory system, lung and chest wall.

**Table 2:** Respiratory mechanics

	PEEP (cmH_2_O)						*p* value		
	4			12			Pathology	PEEP	*V*_T_
	*V*_T_								
	8	10	12	8	10	12			
Airway plateau pressure (cmH_2_O)							<0.001	<0.001	<0.001
ARDS patients	18.6 ± 3.6	21.7 ± 4.3	23.4 ± 3.3	26.5 ± 2.7	29.2 ± 2.7	31.9 ± 2.6			
Control patients	13.3 ± 2.1	14.7 ± 2.7	16.0 ± 3.1	21.2 ± 2.3	22.7 ± 2.6	24.3 ± 3.3			
Respiratory system compliance (ml/cmH_2_O)							<0.001	0.36	0.40
ARDS patients	9.6 ± 4.6	9.9 ± 5.6	10.2 ± 4.4	9.0 ± 4.0	9.4 ± 4.2	9.6 ± 4.2			
Control patients	16.5 ± 3.4	17.9 ± 4.8	19.0 ± 6.9	16.0 ± 3.6	17.1 ± 4.3	17.6 ± 4.5			
Lung compliance (ml/cmH_2_O)							<0.001	0.56	0.43
ARDS patients	11.5 ± 6.0	11.6 ± 5.5	12.0 ± 5.5	10.2 ± 4.5	10.9 ± 5.1	11.3 ± 4.9			
Control patients	21.8 ± 4.6	23.8 ± 6.2	25.5 ± 8.0	22.4 ± 6.0	23.4 ± 8.3	24.2 ± 7.9			
Chest wall compliance (ml/cmH_2_O)							0.49	0.60	0.63
ARDS patients	76.3 ± 29.6	79.2 ± 35.0	74.5 ± 26.3	122.6 ± 113.0	81.4 ± 30.3	73.8 ± 52.1			
Control patients	99.5 ± 99.1	85.6 ± 55.8	98.2 ± 69.1	85.5 ± 61.6	90.1 ± 55.9	94.4 ± 69.1			
End-expiratory lung volume (ml)							<0.001	<0.001	0.97
ARDS patients	211.0 ± 116.1	216.5 ± 115.2	216.7 ± 117.7	346.2 ± 177.6	361.2 ± 185.6	374.2 ± 183.9			
Control patients	386.2 ± 156.6	384.7 ± 151.7	386.9 ± 156.4	619.2 ± 230.2	623.1 ± 219.9	622.1 ± 207.2			

*ARDS* acute respiratory distress syndrome

Values are mean ± SD. Two statistical analyses are reported: a three-way analysis of variance to test the effects of the presence of the disease, the level of PEEP and the level of V_T_/IBW and a post hoc Bonferroni’s test analysis for the comparison between subgroups

In both groups, the compliance of respiratory system and that of the lung were significantly related to end-expiratory lung volume (*r*^2^ = 0.49, *p* < 0.01; *r*^2^ = 0.44, *p* < 0.01) and (*r*^2^ = 0.43, *p* < 0.01; *r*^2^ = 0.34, *p* < 0.01, respectively) (see Additional file 1: Figures S4, S5, S6, S7). Compliance of respiratory system was significantly related to the age of the patients in ARDS group (*r*^2^ = 0.64, *p* < 0.005) but only showed a tendency toward significance in the control group (*r*^2^ = 0.35, *p* = 0.007) (see Additional file 1: Figures S8, S9).

EELV was significantly lower in ARDS patients and increased in both groups after increasing the level of PEEP.

### Stress and strain

**Fig. 2:**
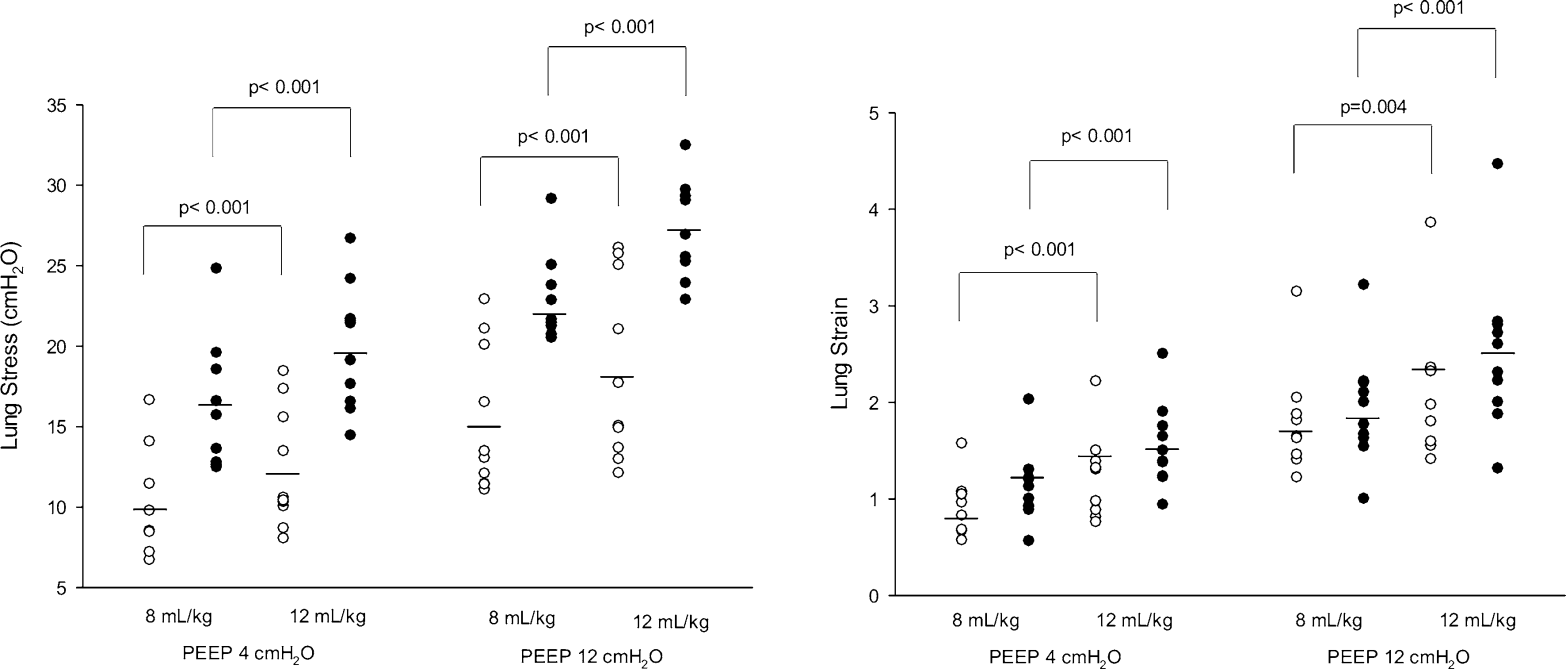
Lung stress (*left panel*) and strain (*right panel*) at 8 and 12 ml/kg of ideal body weight in control and ARDS patients. Individual values are reported for ARDS (*solid circle*) and control group (*open circle*), and *black solid lines* represent mean values of each group

**Fig. 3:**
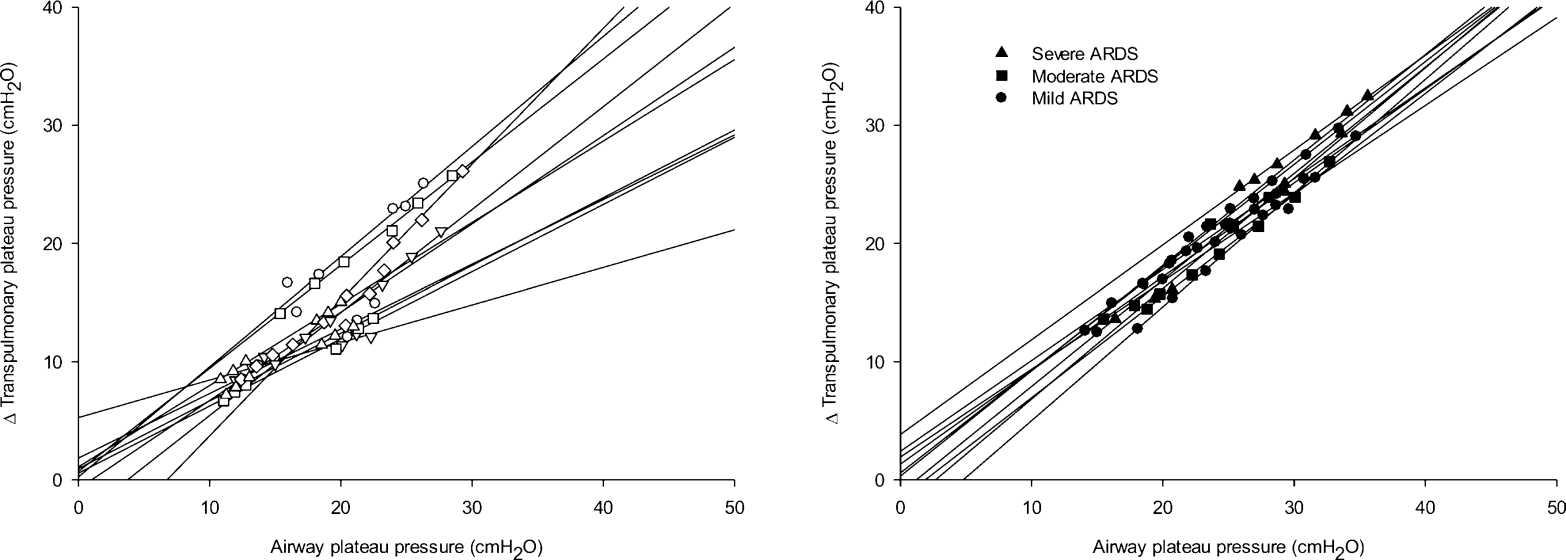
Relationship between the changes in transpulmonary plateau pressure and airway plateau pressure in control (*left panel*) and ARDS patients (*right panel*). In both groups, the solid lines represent the relationship observed in each individual subject in the six experimental conditions (three different tidal volumes 8, 10 and 12 ml/kg of ideal body weight at two different levels of PEEP 4 and 8 cmH_2_O)

The lung stress and strain were significantly higher in ARDS patients as compared to control subjects and increased after increasing the level of PEEP and tidal volume (Table 3). ARDS patients presented greater changes in transpulmonary pressure and strain as compared to control subjects (Fig. 2). However, there was a large data overlap in the two groups. For a similar airway plateau pressure, there was a huge difference in transpulmonary pressures (Fig. 3).

**Table 3:** Lung stress, strain and specific lung elastance

	PEEP (cmH_2_O)						*p* value		
	4			12			Pathology	PEEP	*V*_T_
	V_T_								
	8	10	12	8	10	12			
Lung stress (cmH_2_O)							<0.001	<0.001	<0.001
ARDS patients	16.0 ± 4.0	17.7 ± 3.8	19.9 ± 3.7	22.9 ± 2.8	24.6 ± 3.0	27.2 ± 2.8			
Control patients	9.9 ± 3.1	10.9 ± 3.0	12.2 ± 3.2	15.3 ± 4.4	16.8 ± 4.6	18.4 ± 5.5			
Lung strain							0.013	<0.001	0.030
ARDS patients	1.13 ± 0.39	1.32 ± 0.43	1.53 ± 0.46	1.94 ± 0.60	2.19 ± 0.70	2.39 ± 0.86			
Control patients	0.94 ± 0.30	1.08 ± 0.36	1.23 ± 0.45	1.80 ± 0.56	1.97 ± 0.67	2.13 ± 0.74			
Specific lung elastance (cmH_2_O)							<0.001	0.024	0.586
ARDS patients	16.22 ± 7.8	14.21 ± 6.2	13.81 ± 4.8	13.23 ± 4.9	12.41 ± 4.7	12.36 ± 4.9			
Control patients	11.24 ± 3.3	10.62 ± 2.3	10.46 ± 2.4	8.98 ± 2.2	9.04 ± 1.8	9.21 ± 1.8			

*ARDS* acute respiratory distress syndrome

Values are mean ± SD. Two statistical analyses are reported: a three-way analysis of variance to test the effects of the presence of the disease, the level of PEEP and the level of VT/IBW and a post hoc Bonferroni’s test analysis for the comparison between subgroups

The ratio between the lung stress and strain (i.e., the specific lung elastance) was significantly higher in ARDS patients (Table 3).

Changes in lung stress as function of changes in airway driving pressure during the PEEP trial in the individual patients are reported in Figure S10

## Discussion

The primary findings of this study are that (1) children with ARDS presented a significantly lower compliance of the respiratory system and of the lung compared with control subjects; (2) chest wall compliance was similar in the two groups; (3) compliance of the lung and chest wall was not affected by the changes in PEEP or tidal volume; (4) stress and strain were significantly higher in ARDS compared with control subjects; and (5) the specific lung elastance decreased with PEEP

Up to 30 % of all children admitted in pediatric intensive care are intubated and mechanically ventilated, mainly for respiratory and cardiovascular disorders [5, 6]. The optimal ventilator management of ARDS is still unresolved because the “adult” ventilatory strategies have rarely been tested in randomized pediatric setting [11–13] and conclusive link between use of large tidal volume and mortality has not been demonstrated [1, 23].

Limited data reported the alteration of respiratory mechanics in children with ARDS [16–18, 24, 25]. Thus, identifying these changes in respiratory mechanics in children with ARDS could provide useful information for the possible detrimental effects of mechanical ventilation [26, 27].

### Compliance of respiratory system

Due to the increase in number and alveoli size during the adolescent, the compliance of respiratory system and of the lung significantly increased with increasing age [28–34]. On the contrary due to the rapid ossification and changes in rib cage configuration, the chest wall compliance decreased within the first years [24]. However, the chest wall is nearly three times more elastic compared with normal lung [24], and thus, the chest wall contributes only 30–35 % to the total respiratory system [35].

In order to correctly compare the data of respiratory mechanics reported by the different studies, it is essential to consider the patients’ age, the technique applied (static or dynamic, inspiratory or expiratory pressure volume curve), the presence of sedation or anesthesia, the size of tidal volume and the level of applied PEEP [32, 36]. In our study evaluating the respiratory mechanics during inspiration in static conditions, the control group (i.e., healthy children) had an average compliance of the respiratory system of 17.4 ± 4.3 (ranging between 14.7 and 20.6 ml/cmH_2_O). Similar results were reported in previous studies, in a group of children sedated and mechanically ventilated prior the surgery, in which the compliance of the respiratory system ranged between 20 and 30 ml/cmH_2_O [28, 35]. Applying an automatic computation with the single breath occlusion technique available in modern ventilators, it has been reported an average compliance of respiratory system of 22.8 ± 12 ml/cmH_2_O [37]. In a group of younger patients with a mean age from 1 to 25 months, the compliance of respiratory system was significantly lower compared with published data with an average value of 4 ml/cmH_2_O [36].

The presence of lung disease has been reported to significantly reduce the functional residual capacity compared with healthy subjects [16, 38]. The increase in functional residual capacity due to the application of PEEP is generated by the recruitment of new lung unit and by the over-distension of already open lung unit, and consequently, the final effect will depend on the balance of these two. Numa et al. [16] found in restrictive patients of 2.0 years old a mean functional residual capacity of 14.1 ± 1.9 ml/kg compared with 26.4 ± 1.8 ml/kg in healthy subjects. In the present study, children with ARDS had a significantly lower functional residual capacity compared with healthy subjects (10.4 [9.1–14.3] ml/kg compared with 16.6 [11.7–24.6] ml/kg). As the compliance of respiratory system is partially related to the amount of lung aeration (i.e., end-expiratory lung volume), compliance was significantly lower in ARDS compared with the healthy subjects. However, compliance did not change with the amount of tidal volume and/or the level of PEEP, suggesting a mixed effect of possible simultaneous lung recruitment and over-distension. Fletcher et al. [36] found a significant increase in the compliance of respiratory system when tidal volume was increased from 3.3 to 9.3 ml/kg only in anesthetized children. In children with non-cardiogenic pulmonary edema, the increase in PEEP from 0 to 18 cmH_2_O improved the compliance of respiratory system in only 60 % of the patients [18].

### Lung and chest wall compliance

To better understand how the respiratory mechanics are affected, we have considered the lung and chest wall compliance by computing the transpulmonary pressure. The transpulmonary pressure is the distending force of the lung, and it was computed as the difference in the changes in airway pressure and esophageal pressure. Esophageal pressure was recorded with an esophageal balloon, which has been shown to accurately reflect the pleural pressure in previous studies [29, 30, 32, 33]. Comparing similar anthropometric features with our study, Nisbet et al. [28] found that lung compliance ranged from 30 to 40 ml/cmH_2_O, slightly higher compared with the present data. Similarly, Ingimarsson et al. [32] reported that in muscle paralyzed healthy children the compliance of lung averaged 3.3 ± 0.7 ml/cmH_2_O/kg. In the present study, the lung compliance was significantly higher in healthy children compared with ARDS 1.5 ± 0.5 ml/cmH_2_O/kg vs 0.7 ± 0.1 ml/cmH_2_O/kg and did not change with PEEP or tidal volume.

In healthy children, the chest wall compliance is usually higher compared with lung compliance promoting the tendency for lung to collapse at low lung volume, being the rib cage relatively ineffective for opposing the inward recoil of the lungs [39, 40]. A significantly higher chest wall compliance was found in patients with neuromuscular disorders predisposing these subjects to development of atelectasis and hypoxemia. Nisbet et al. [28] reported in children during general anesthesia a chest wall compliance ranging from 70 to 100 ml/cmH_2_O. Similarly, data were found in the present study without any difference between control and ARDS subjects. The lack of difference in chest wall compliance between ARDS and control group was mainly due to the presence of only pulmonary ARDS which has been mainly associated with a reduction only in the lung compliance [19].

### Stress and strain

Similarly, to adult patients with and without ARDS the changes in airway pressure were poorly related to changes in transpulmonary pressure which is the distending force of the lung (i.e., the stress) [19]. Thus assuming a “safe” limit of 30 cmH_2_O of airway pressure the resulting transpulmonary pressure can vary from 27.1 to 23.8 cmH_2_O passing from a safe zone to a probably unsafe zone. Also the tidal volume normalized for the predicted body weight in both groups due to the unpredictable reduction in functional residual capacity produced significant difference in the lung “strain”. Compared with lung compliance which did not change with PEEP, the stress and strain significantly increased, suggesting that they could be used as better indicator for possible ventilator-induced lung injury when setting mechanical ventilation. On the contrary, the lung stress, although related to airway driving pressure, could not be predicted by the driving pressure. In fact, for an airway driving pressure between 14 and 16 cmH_2_O the lung stress ranged between 13 and 25 cmH_2_O.

In adult patients with or without ARDS, similar changes in transpulmonary pressure cause similar changes in lung gas volume, suggesting similar specific lung elastance [19]. On the contrary, in children with ARDS the specific lung elastance was significantly higher compared with control group. This suggests not only that in children with ARDS there is a decrease in lung gas volume but that the ventilated tissue presents different structural characteristics. Inflammations, surfactant depletion/alterations and edema may explain these different tissue behaviors compared with adults in which specific lung elastance was similar between ARDS and control groups.

### Limitation

Possible limitations of this study are: (1) the relatively few number of enrolled patients; (2) the absence of any patient with an extrapulmonary ARDS; and (3) the strain computed without taking into account the recruitment during inspiration because it was assumed that the similar amount of pulmonary units is open at end inspiration and expiration.

## Conclusions

In conclusion, in children with ARDS the lung stress cannot be predicted from the airway pressure and the tidal volume normalized for the body weight can produce different amounts of lung strain. Thus, an ideal respiratory monitoring system in children with mild-to-moderate ARDS should provide the measurement of stress and strain.

## Additional file

10.1186/s13613-016-0113-0 Respiratory mechanics and lung stress/strain in children with acute respiratory distress syndrome.

## Abbreviations

ARDS: acute respiratory distress syndrome
PEEP: positive end-expiratory pressure
EELV: end-expiratory lung volume
