# Supplementary material for: Respiratory mechanics and lung stress/strain in children with acute respiratory distress syndrome
Source: Ann Intensive Care. 2016 Feb 5;6:11. doi: 10.1186/s13613-016-0113-0 (PMC4742456; doi:10.1186/s13613-016-0113-0)
Supplement: Supplementary file 1 — 10.1186/s13613-016-0113-0 Respiratory mechanics and lung stress/strain in children with acute respiratory distress syndrome. [file 13613_2016_113_MOESM1_ESM.pdf]

RESPIRATORY MECHANICS AND LUNG STRESS / STRAIN IN CHILDREN WITH ACUTE RESPIRATORY DISTRESS SYNDROME

Davide Chiumello, MD[1,2]; Giovanna Chidini MD[3], Edoardo Calderini[3], Andrea Colombo[2], Francesco Crimella[2], Matteo Brioni[2]

[1] Dipartimento di Anestesia, Rianimazione (Intensiva e Subintensiva) e Terapia del Dolore, Fondazione IRCCS Ca' Granda - Ospedale Maggiore Policlinico, Milano, Italy

[2] Dipartimento di Fisiopatologia Medico-Chirurgica e dei Trapianti, Università degli Studi di Milano, Italy

[3] Terapia Intensiva Pediatrica, Dipartimento di Anestesia e Rianimazione, Fondazione IRCCS Ca Granda Ospedale Maggiore Policlinico, Milano, Italy

Figure S1. Schematic representation of the lung volume changes observed during the different steps of the experimental protocol.

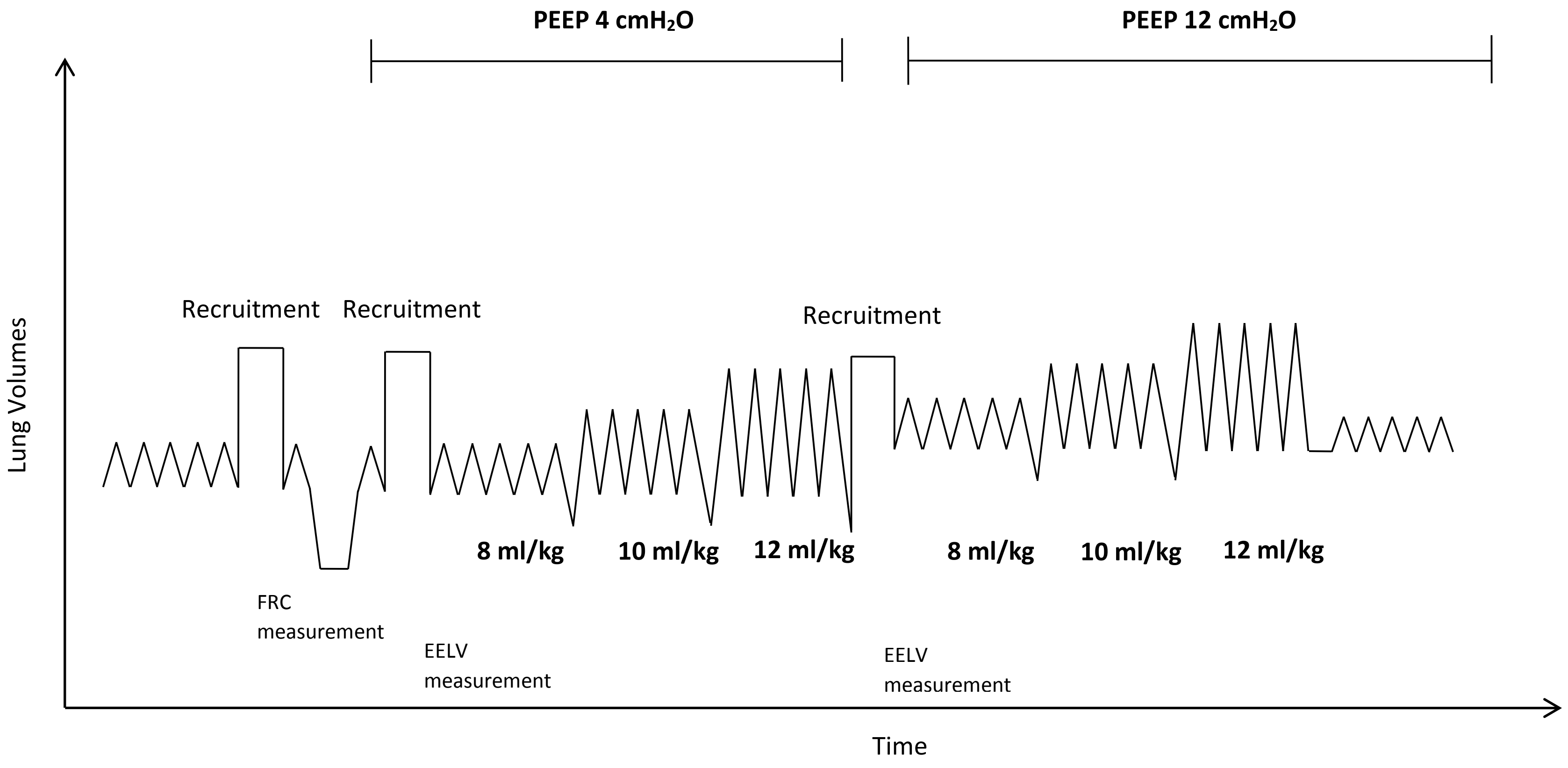

Figure S2. Linear regression between functional residual capacity and age in ARDS patients (y=98.05+1.32x, r2=0.71, p=0.002).

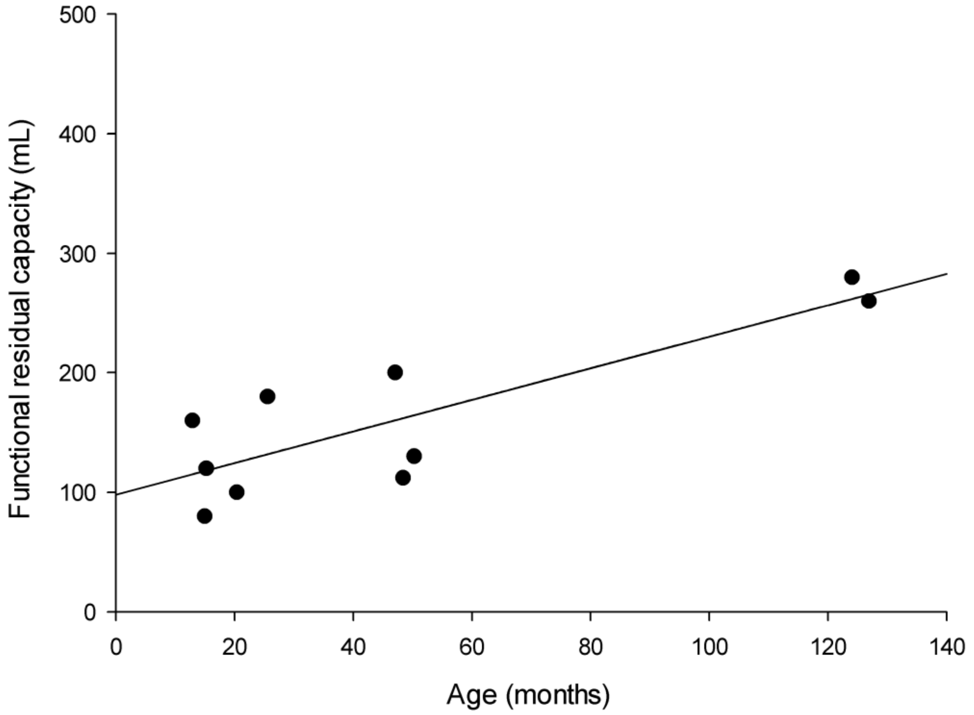

Figure S3. Linear regression between functional residual capacity and age in control patients (y=176.81+1.86x, r2=0.49, p=0.02).

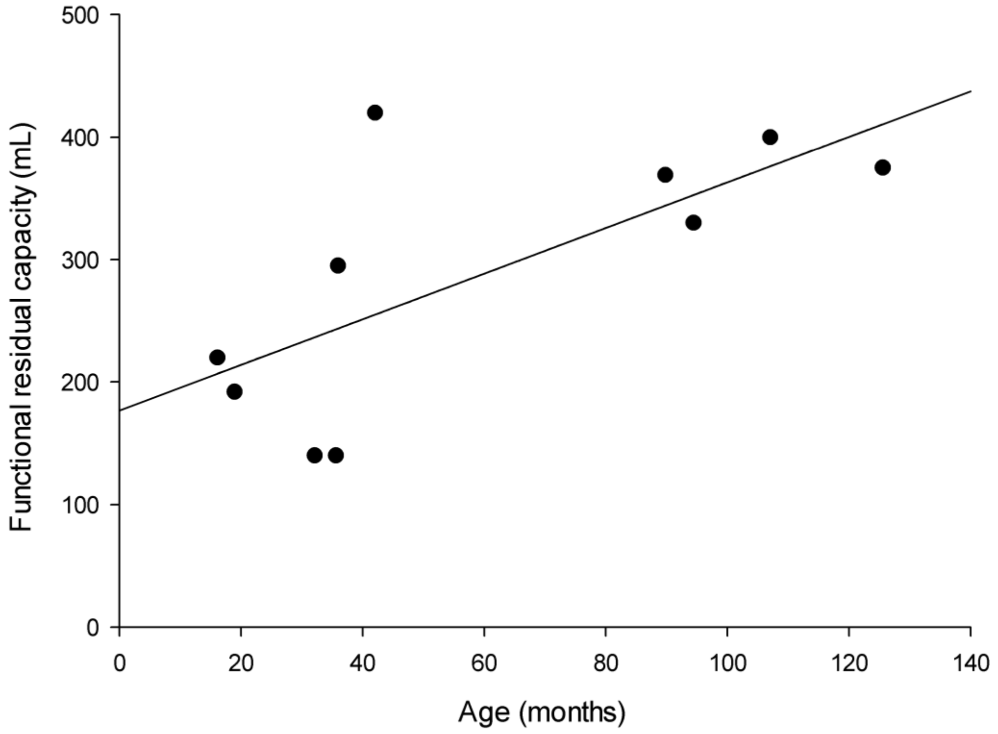

Figure S4. Linear regression between respiratory system compliance and end expiratory lung volume in ARDS patients (y=4.38+0.01x, r2=0.49, p<0.001).

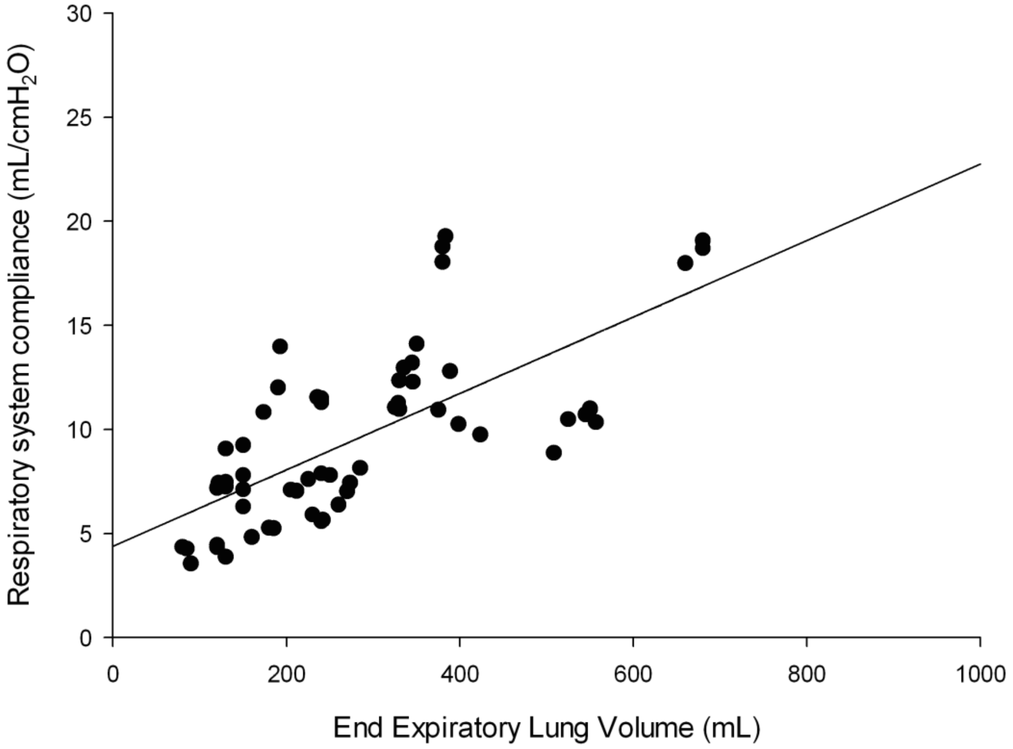

Figure S5. Linear regression between lung compliance and end expiratory lung volume in ARDS patients (y=5.04+0.02x, r2=0.44, p<0.001).

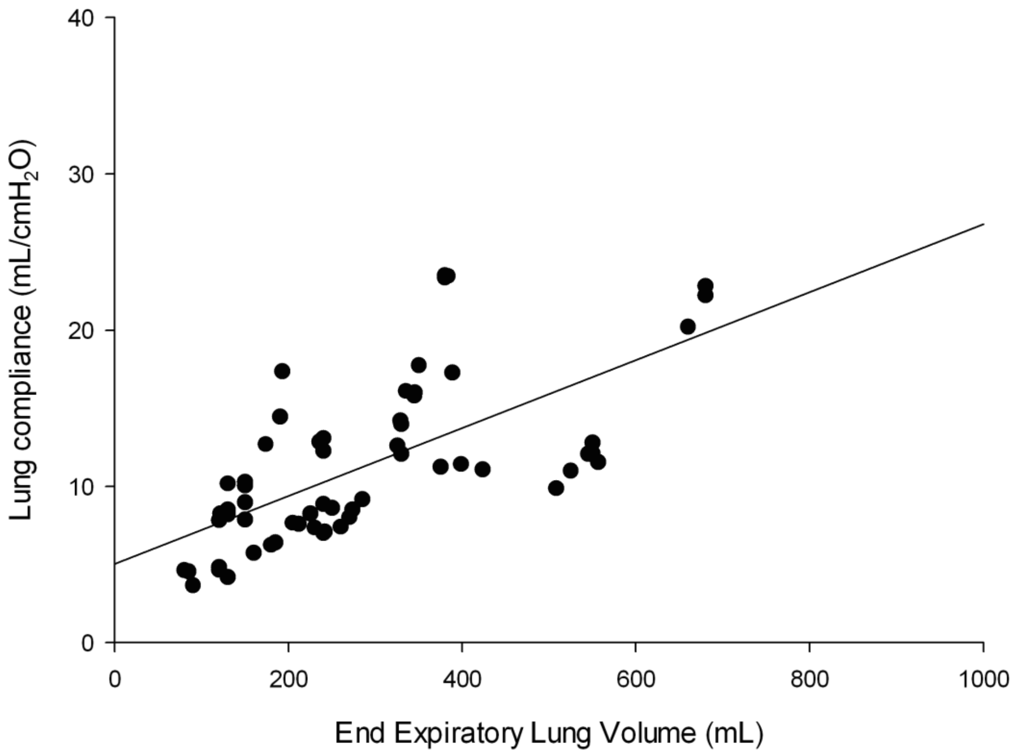

Figure S6. Linear regression between respiratory system compliance and end expiratory lung volume in control patients (y=10.63+0.01x, r2=0.43, p<0.001).

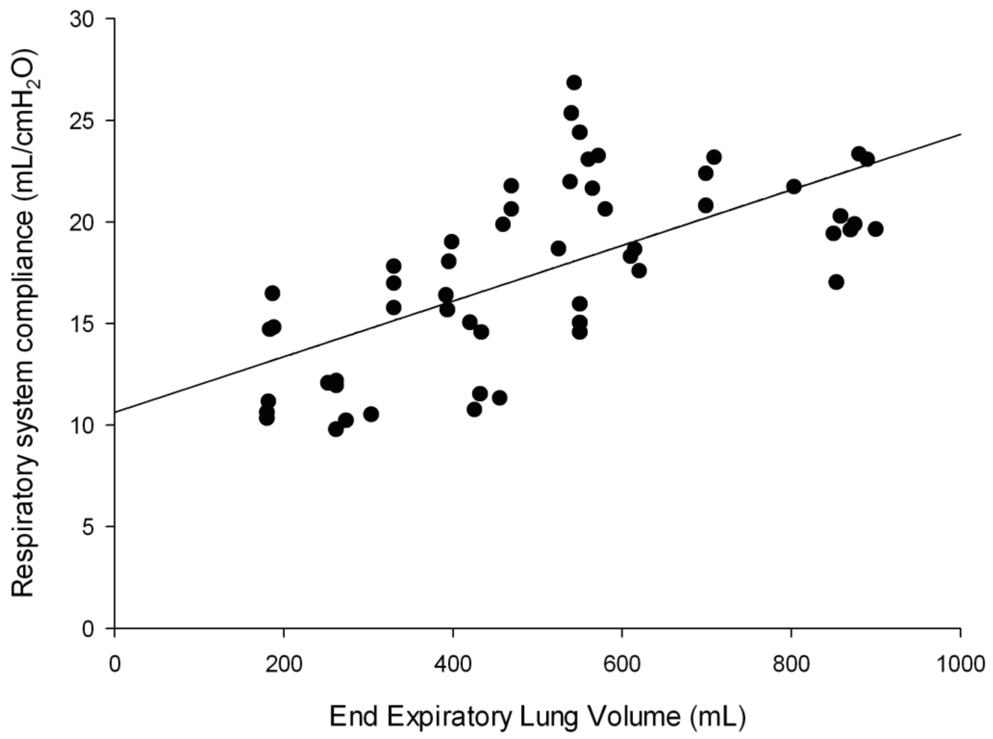

Figure S7. Linear regression between lung compliance and end expiratory lung volume in control patients (y=13.78+0.01x, r2=0.34, p<0.001).

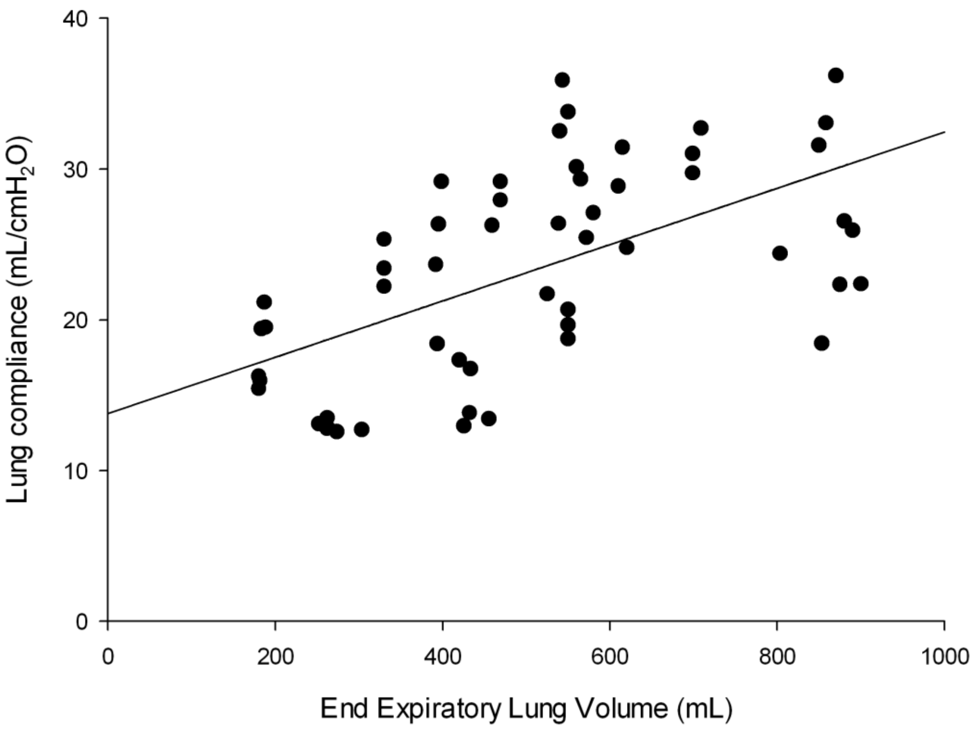

Figure S8. Linear regression between respiratory system compliance and age in ARDS patients (y=7.87+0.09x, r^2=0.64, p=0.005).

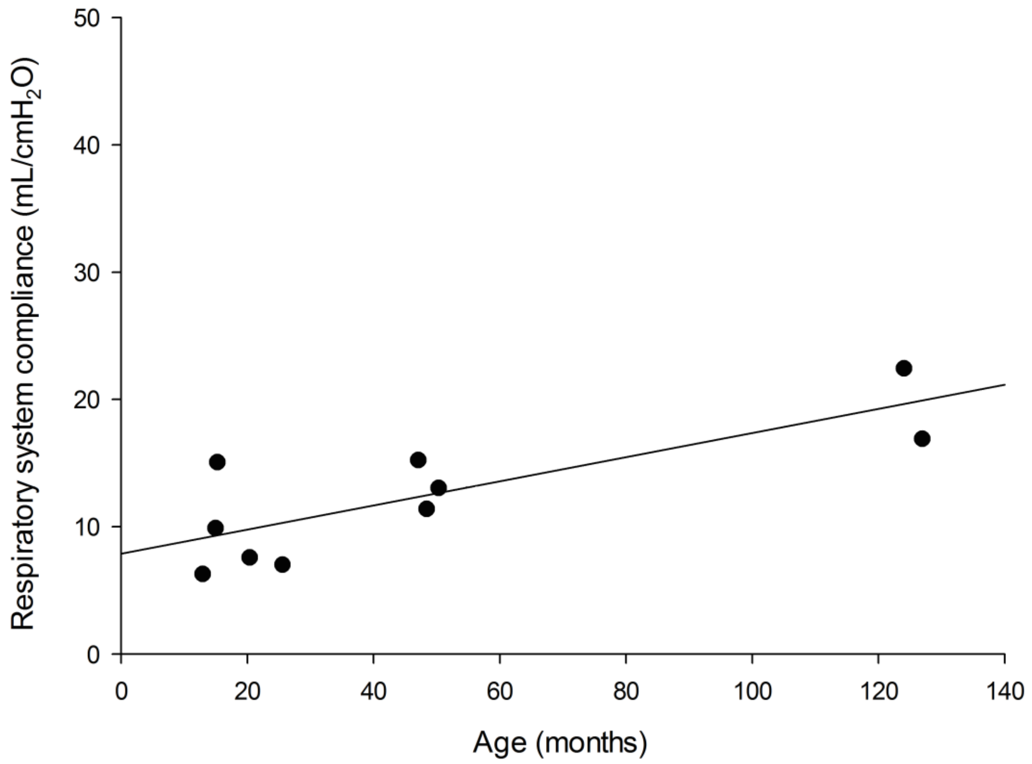

Figure S9. Linear regression between respiratory system compliance and age in control patients ($y=17.79+0.15x$, $r^2=0.35$, $p=0.071$).

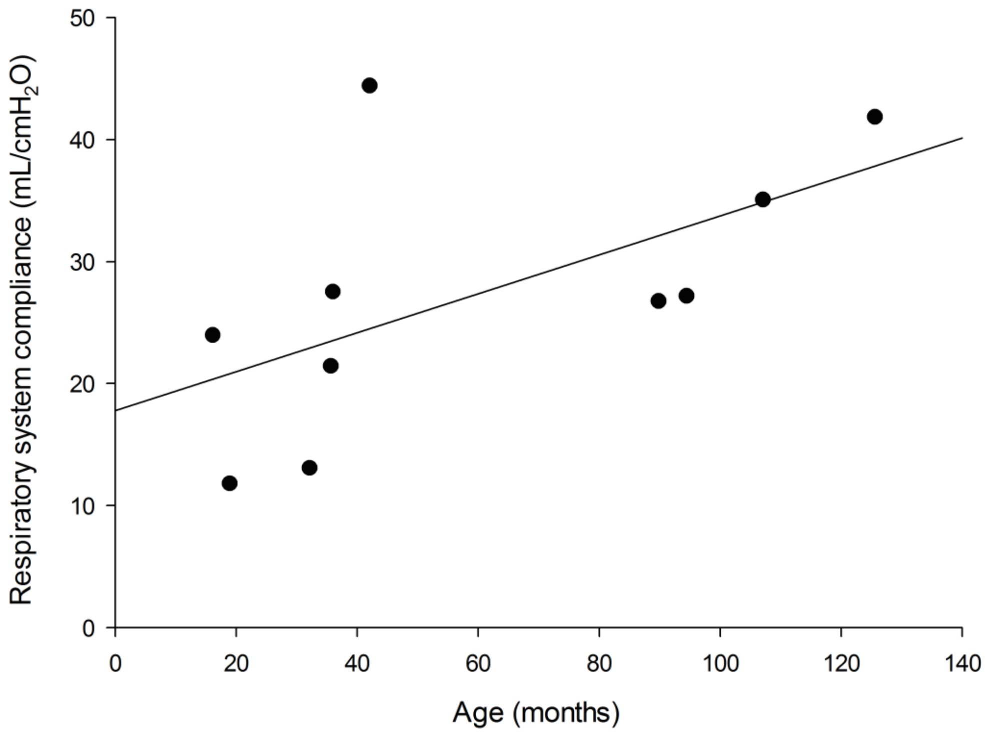

Figure S10. Linear regression between airway driving pressure and lung stress considering both ARDS and control group (y=3.135+1.058x, r^2=0.689, p<0.001).

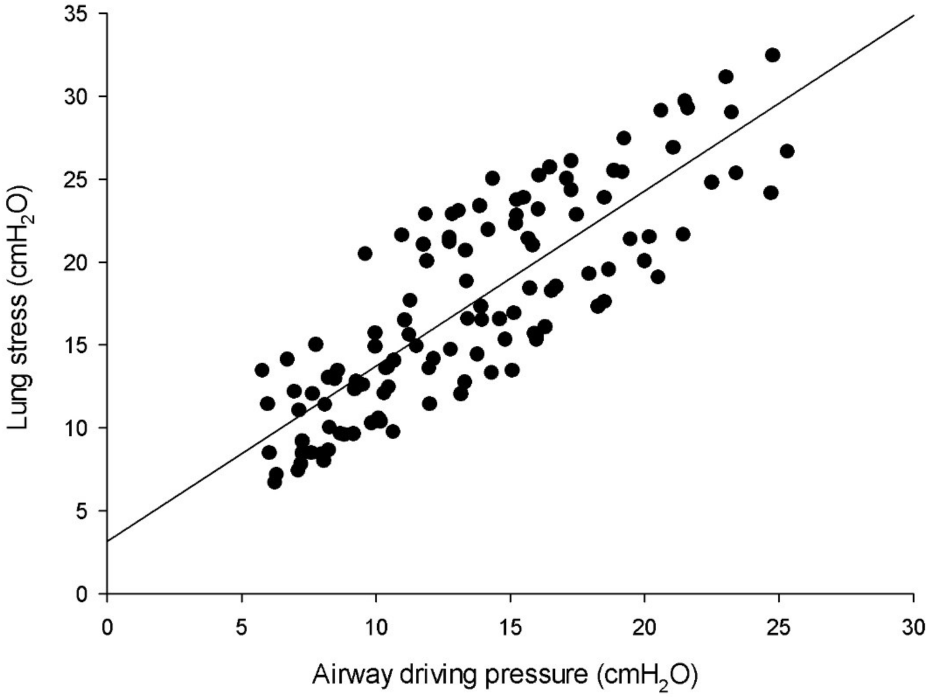

Figure S11. Linear regression between lung specific elastance (mean of the determinations at different PEEP ad Vt) and patient's age (y=11,538+0,00304x, r^2=0.00126 p=0.882)

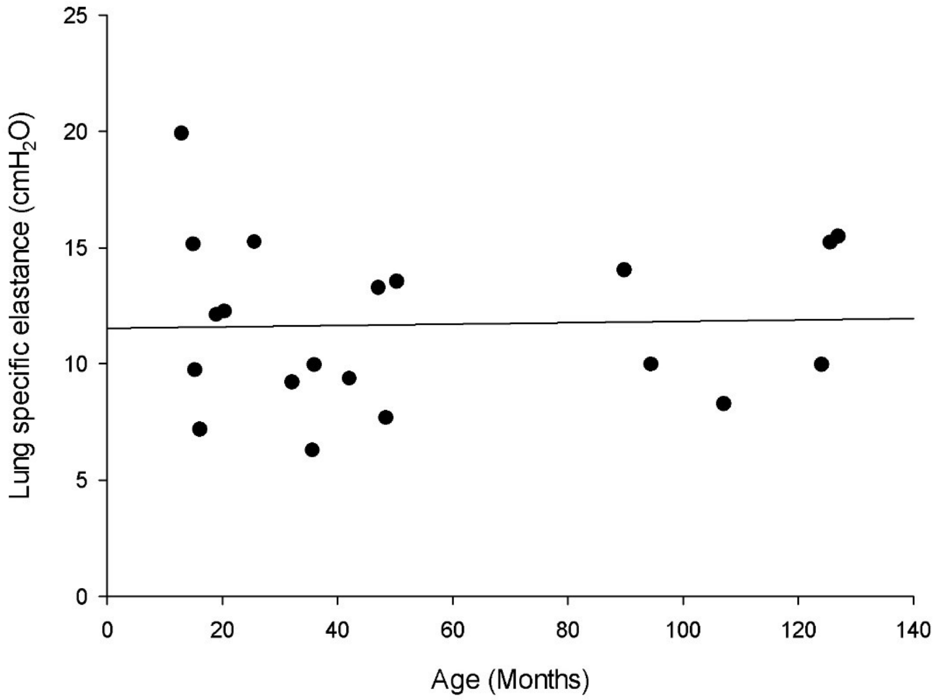

Table S1. Comparison between patients with mild and moderate-to-severe ARDS.

Variable	ARDS patients (N=10)	Mild ARDS (N=6)	Moderate to severe ARDS (N=4)	P value
**Age (Months)**	36 [15 – 50]	23 [15 – 48]	49 [31 – 89]	0.476
**FRC (mL)**	145[112 – 200]	140 [112 – 180]	165 [105 – 230]	0.853
**EELV at PEEP 4 cmH_2O (mL)**	191 [139 – 280]	191 [140 – 213]	210 [125 – 311]	0.879
**EELV at PEEP 12 cmH_2O (mL)**	324 [286 – 387]	324 [286 – 365]	342 [229 – 435]	0.814
**Lung specific elastance (cmH_2O) ***	13.4 [10.0 – 15.3]	11.1 [9.7 – 15.3]	14.4 [13.4 – 15.3]	0.437
**Ers at PEEP 4 cmH_2O (cmH_2O/L) ***	103.4 [82.4 – 142.5]	103.3 [82.4 – 135.6]	115.0 [81.1 – 195.5]	0.539
**Ecw at PEEP 4 cmH_2O (cmH_2O/L) ***	12.9 [10.9 – 15.3]	12.7 [10.9 – 15.3]	12.9 [10.7 – 22.0]	0.914
**El at PEEP 4 cmH_2O (cmH_2O/L) ***	93.2 [68.5 – 120.2]	93.2 [68.5 – 120.2]	95.3 [69.3 – 174.6]	0.531
**Ers at PEEP 12 cmH_2O (cmH_2O/L) ***	113.5 [92.5 – 144.5]	111.6 [82.8 – 138.1]	122.7 [96.7 – 191.1]	0.382
**Ecw at PEEP 12 cmH_2O (cmH_2O/L) ***	12.1 [8.5 – 18.7	11.9 [8.3 – 19.2]	13.9 [9.6 – 17.9]	0.746
**El at PEEP 12 cmH_2O (cmH_2O/L) ***	101.4 [84.0 – 127.8]	100.7 [63.6 – 127.8]	108.0 [87.1 – 173.2]	0.313

FRC: functional residual capacity; EELV: end-expiratory lung volume; PEEP: positive end-expiratory pressure; Ers: respiratory system elastance; El: lung elastance; Ecw: chest wall elastance.

*mean of the value measured at Vt 8-10-12 mL/kg

Statistical analysis: student's t-test, Mann-Whitney Rank Sum Test, as appropriate.
